# O13 - Regional differences in sensitisation to ragweed in Croatian children are not associated solely with pollen concentration

**DOI:** 10.1186/2045-7022-4-S1-O13

**Published:** 2014-03-14

**Authors:** Marija Perica, Adrijana Miletić Gospić, Ana Večenaj, Davor Plavec, Mirjana Turkalj

**Affiliations:** 1 Children's Hospital Srebrnjak, Zagreb, Croatia

## Introduction

FP7 project ATOPICA (*Atopic diseases in changing climate*, *land use and air quality*) supported by EU Grant agreement NO: CP 282687 explores the combined pan-European impact of changes in climate, land use and air pollution on allergen pollen-induced diseases with an accent on atopy due to ragweed sensitization. Sensitization to ragweed pollen correlates with levels of airborne pollen concentration in environment, but can be enhanced by other environmental factors such as air pollution.

## Material and Methods

Cohort of 3588 children, aged 4-10 years, was recruited from 3 regions of Croatia differing in airborne pollen concentrations (Slavonia, Zagreb and surrounding end Dalmatia). Each participant underwent skin prick test (SPT) to the standard set of aeroallergens. For each region, pollen concentrations and air quality data were gathered from authorized institutions.

## Results

A total of 990 children were sensitized to one or more aeroallergens. Prevalence of ragweed sensitization was 14.84 % in Zagreb area, 14.26 % in Slavonia and 1.53 % in Dalmatia. Comparing the highest pollen concentrations during ragweed pollinating period among 3 regions, Dalmatia has the lowest concentration of ragweed pollen of 30-40 grains/m^3^, while Zagreb measures 250-300 grains/m^3^ and Slavonia 700-1000 grains/m^3^ per 24 hours. Analysis of sensitization in two age groups (4-6 and 7-10 years) reveals higher prevalence of ragweed, birch and D. pteronyssinus sensitization as well as double sensitization (birch and ragweed) in older age group for all 3 regions. Sensitization to above allergens was more prevalent in male participants.

## Conclusions

Although region of Slavonia measures highest ragweed pollen concentrations, the equal portion of sensitized children was found in Zagreb and surrounding areas having 3 times lower concentrations of ragweed pollen. However, as a largest city in Croatia with a number of industrial zones in its surrounding, air pollution in Zagreb is highly present. Detailed statistical analysis on the impact of air pollution on sensitization rate is in progress.

